# Patient-Derived Organoids as a Platform to Decipher and Overcome Radioresistance: From the Tumor Microenvironment to Radiosensitizer Discovery

**DOI:** 10.3390/curroncol32120680

**Published:** 2025-12-01

**Authors:** Dashan Yin, Xiujuan Hong, Xiaoqi Wang, Wenjia Ding, Chenli Wang, Jin Qian, Yi Zhou, Chuan Sun, Zhibing Wu

**Affiliations:** 1 School of Medicine, Zhejiang University, Hangzhou 310058, China; 22318702@zju.edu.cn (D.Y.); 12418722@zju.edu.cn (X.H.); 0624090@zju.edu.cn (X.W.); wenjia@zju.edu.cn (W.D.);; 2 Second Clinical Medical College, Zhejiang Chinese Medical University, Hangzhou 310053, China

**Keywords:** patient-derived organoids (PDOs), radioresistance, radiosensitizers, radiotherapy (RT), tumor microenvironment (TME), cancer stem cells (CSCs), DNA damage response (DDR), hypoxia and redox homeostasis

## Abstract

Patient-derived organoids (PDOs) preserve patient genotypes and 3D architecture, offering a useful platform to investigate mechanisms of radioresistance and test radiosensitizers. We outline an end-to-end workflow—model establishment, multi-omics profiling, pharmacologic screening, and in vivo confirmation—and spotlight immune-competent, vascularized, and organ-on-chip formats. PDOs reveal actionable mechanisms across DNA damage response, hypoxia–metabolic and immune remodeling, and radiation-induced senescence, enabling rational radiosensitizer selection. Paired tumor–normal organoids concurrently gauge efficacy and normal tissue toxicity, refining the therapeutic index. Remaining gaps (incomplete microenvironment, fractionation modeling, and standardization) are being addressed via reporting standards and co-clinical studies, positioning PDOs to support precision radiotherapy.

## Simple Summary

Radiotherapy is one of the most effective treatments for cancer, but its success is often limited by tumor radioresistance and the complex interactions within the tumor microenvironment. Patient-derived organoids are miniature, three-dimensional models grown from a patient’s own tumor that closely mimic the biology and treatment response of real tumors. These organoids provide a promising tool to study why some tumors resist radiation and to test strategies that make radiotherapy more effective. This review summarizes how organoids are used to explore the mechanisms of radioresistance—such as DNA repair, tumor metabolism, immune remodeling, and radiation-induced senescence—and discusses how integrating organoid models with immune and vascular components could accelerate the discovery of personalized radiosensitizers and improve cancer treatment outcomes.

## 1. Introduction

Radiotherapy (RT) treats roughly half of all cancer patients, yet durable control is often limited by radioresistance. Mechanisms span enhanced DNA damage repair, cancer stem cell plasticity, hypoxia-driven redox and metabolic reprogramming, and immune remodeling within the tumor microenvironment (TME) [1].

Patient-derived organoids (PDOs) preserve tumor histology, genomics, and intratumoral heterogeneity, offering a tractable platform to interrogate resistance biology and functionally test therapies in a patient-proximal manner [2,3]. Emerging evidence indicates that organoid responses can mirror clinical treatment sensitivity, including to radiation in specific settings. In parallel, advanced co-cultures and organ-on-a-chip systems enable incorporation of stromal, immune, and vascular components and support concurrent assessment of normal tissue toxicity—key for defining a therapeutic window in radiosensitizer development [4].

This review provides a concise synthesis of how PDO platforms are being applied to (i) map cellular and molecular determinants of radioresistance; (ii) anticipate RT response at the individual-patient level; and (iii) guide rational, mechanism-based radiosensitization strategies—including materials-enabled approaches—without pre-empting results or conclusions.

## 2. Patient-Derived Tumor Organoids: Establishment and Application

Organoid technology began in 2009, when Lgr5^+^ intestinal stem cells were used to generate self-organizing small-intestinal organoids with crypt–villus architecture, formally introducing the concept of the “organoid” [5]. Subsequent advances included human colon organoid cultures (2011) and the first colorectal cancer (CRC) organoid model reported, which preserved the mutational landscape of primary tumors [6]. In parallel, a living biobank of CRC organoids was established, bringing patient-specific drug sensitivity testing into practical use [7].

PDOs are established through a standardized process beginning with the acquisition of fresh patient-derived tumor tissue from surgical resections or biopsies. After removal of non-epithelial tissue, samples are mechanically and enzymatically dissociated to small clusters or single cells, cleared of debris, and embedded in a 3D extracellular matrix hydrogel (e.g., Matrigel, Geltrex, and tissue-specific or synthetic matrices). Over days to weeks, cells self-organize into 3D structures that retain key histologic and genetic features of the source tumor. Across PDO subtypes, growth factor formulations vary slightly. Organoids are expanded by mechanical or enzymatic passaging and re-embedding, and can be cryopreserved to create living biobanks that preserve intratumoral heterogeneity for disease modeling, drug screening, and patient-proximal therapy testing [8].

PDOs serve as powerful platforms for both mechanistic dissection of carcinogenesis and therapeutic biomarker discovery. In a Barrett’s esophagus mouse model, organoids derived from Cck2r^+^ cardia progenitor cells revealed that mutant p53 promotes direct progression to dysplasia, bypassing the typical metaplastic stage. These organoids exhibited enhanced self-renewal, increased resistance to DNA-damaging agents such as N-methyl-N-nitrosourea (MNU), and retained dysplastic features following orthotopic transplantation [9]. In parallel, another study using breast and lung cancer PDOs stratified by TP53 status demonstrated that combined treatment with talazoparib and temozolomide produced synergistic cytotoxicity exclusively in mutant p53 models. This combination induced sustained DNA double-strand breaks, independent of BRCA1/2 status, and correlated with high PARP1 and mutant p53 co-expression. These findings highlight PDOs as clinically relevant models for linking genotype-specific oncogenic mechanisms with predictive biomarkers for targeted therapy [10].

While PDOs offer a powerful patient-proximal platform, they represent only one component of the broader landscape of preclinical radiotherapy model systems. To contextualize the advantages and limitations of PDOs relative to commonly used 2D monolayer cultures, patient-derived xenografts (PDXs) are commonly employed. A simplified comparative overview is provided in Table 1. This comparison underscores the complementary nature of these systems and clarifies why PDOs occupy a unique intermediate position between reductionist in vitro assays and complex in vivo models.

## 3. Reconstructing the Tumor Microenvironment in Organoids

The tumor microenvironment (TME) refers to the dynamic ecosystem composed of cancer cells and non-malignant stromal, immune, vascular, and extracellular matrix components within and around the tumor mass [11,12,13]. Cancer cells continuously remodel this microenvironment by secreting soluble factors, inducing angiogenesis and establishing systemic immunosuppression, whereas stromal and immune elements in turn shape cancer cell proliferation, survival, and clonal evolution [14,15,16].

### 3.1. Biomimetic ECM and Decellularized Matrices

**Figure 1:**
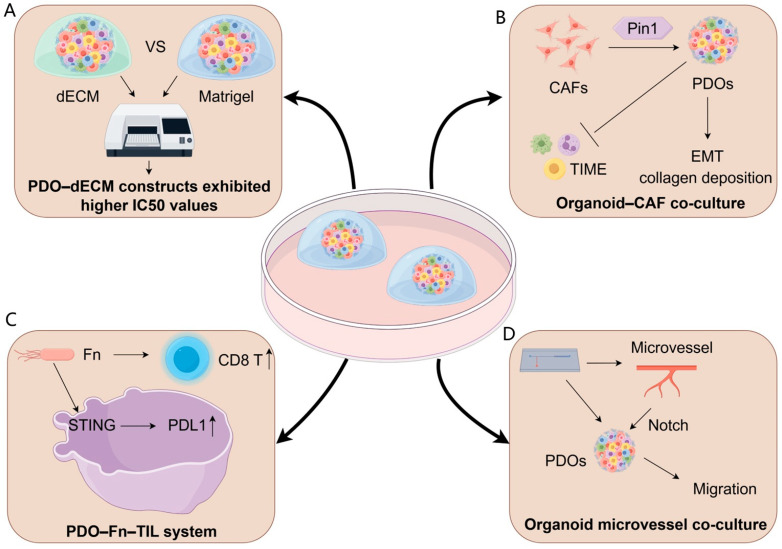
Reconstructing the tumor microenvironment in organoids. (**A**) ECM-derived models: Patient-derived decellularized extracellular matrix (dECM) scaffolds preserve tumor-specific biochemical and mechanical cues, resulting in PDO–dECM constructs that display higher IC_50_ values than Matrigel-grown PDOs, indicating ECM-mediated treatment tolerance. (**B**) Organoid–CAF co-culture: Cancer-associated fibroblasts (CAFs) remodel the TME through Pin1-dependent activation, promoting EMT programs, collagen deposition, and broader immunosuppressive TIME modulation, collectively enhancing PDO invasiveness and treatment resistance. (**C**) PDO–Fn–TIL immune system: Co-culture with Fusobacterium nucleatum (Fn) stimulates STING signaling and upregulates PD-L1 expression in PDOs, accompanied by elevated CD8^+^ T-cell responses, modeling microbe-driven immune modulation. (**D**) Organoid microvessel co-culture: Endothelialized microvessel-on-chip platforms support direct interactions between PDOs and perfusable vascular structures, enabling Notch-mediated migration and providing a physiologically relevant niche to study vascular–tumor crosstalk.

The extracellular matrix (ECM)—a network of structural proteins (e.g., collagens), adhesive glycoproteins (e.g., fibronectin), and glycosaminoglycans (e.g., hyaluronic acid)—regulates tissue morphogenesis and cell identity through biochemical, mechanical, and adhesive cues [17]. In organoid systems, ECM-based hydrogels provide indispensable spatial and mechanical guidance that supports stem cell lineage specification and improves fidelity to in vivo physiology [18,19]. Within the tumor microenvironment (TME), dynamic ECM remodeling promotes growth, invasion, metastasis, and therapy resistance via altered stiffness and signaling [20,21], underscoring the need to recreate native ECM architecture for relevant tumor organoid models [22]. Although Matrigel and other basement membrane extracts (BMEs) are widely used, their murine origin, undefined composition, and batch-to-batch variability limit reproducibility and translation [23]. Decellularized extracellular matrix (dECM) scaffolds preserve native tissue-specific biochemical composition and ultrastructure, thereby providing organotypic cues that cannot be recapitulated by conventional basement membrane extracts such as Matrigel. Recently, Sensi et al. generated a fully patient-derived 3D model of high-grade serous ovarian cancer by combining dECM scaffolds from HGSOC specimens and matched PDOs. The detergent–enzymatic decellularization protocol efficiently removed nuclear material while preserving collagen networks, laminin, glycosaminoglycans, and a tissue-specific matrisome, as demonstrated by SHG imaging, Raman spectroscopy, and mass spectrometry-based proteomics. Importantly, PDOs seeded into HGSOC dECM maintained their histopathological and molecular features and remained viable over prolonged culture. When exposed to paclitaxel or paclitaxel plus carboplatin, PDO–dECM constructs exhibited substantially higher IC50 values than PDOs grown in Geltrex, indicating that patient-derived ECM can attenuate drug sensitivity and better recapitulate in vivo chemoresistance (Figure 1A) [24]. Although this study focused on chemotherapy rather than radiotherapy, it clearly illustrates how patient-derived dECM can modulate treatment response by providing a more realistic mechanical and biochemical microenvironment. Similar dECM–PDO platforms could therefore be leveraged to dissect ECM-driven radioresistance, for example by integrating clinically relevant radiation regimens and readouts of DNA damage repair, clonogenic survival, and inflammatory cell death.

### 3.2. Cancer-Associated Fibroblasts

Cancer-associated fibroblasts (CAFs) are central architects of the tumor microenvironment (TME) and pivotal mediators of radioresistance (Table 2). Co-culturing CRC organoids with patient-matched CAFs reconstructs TME niches, restoring downregulated pro-tumorigenic transcripts (e.g., REG family, DUOX) and reactivating immune-related programs. This confers proliferative advantages, anti-apoptotic capacity, and chemoresistance—highlighting CAF-enriched niches as essential for CRC malignancy [25].

Mechanistically, organoid–CAF co-cultures identify Pin1 as a critical CAF regulator: Pin1-deficient CAFs fail to promote organoid growth/invasion in vitro and suppress collagen deposition/tumor progression in vivo, positioning stromal Pin1 as a therapeutic target (Figure 1B) [26]

Clinically, CAF-derived gene signatures predict radiotherapy outcomes in prostate cancer [27], while pancreatic CAF heterogeneity (myCAFs vs. iCAFs) dictates tumor behavior and treatment response [28].

**Table 2:** Key CAF roles, pathways and interventions implicated in radioresistance.

Study (Year)	Cancer Type	Core CAF Role	Key Pathway(s)	Intervention Strategy
Yang et al., 2023 [29]	ESCC	Collagen I deposition; epithelial–CAF CXCL1 positive feedback	Integrin/FAK–AKT–c-Myc/Chk1; CXCL1/CXCR2–STAT3	Block collagen/FAK; inhibit CXCL1/CXCR2
Meng et al., 2021 [30]	NSCLC	RT-induced senescent CAFs with SASP	SASP → JAK/STAT3	Senolytics (e.g., FOXO4-DRI)
Xun et al., 2025 [31]	TNBC	CAF exosomal circRNA drives autophagy	circFOXO1–miR-27a-3p–BNIP3; autophagy	Block exosome release/uptake; target circFOXO1/miR-27a-3p
Zhang et al., 2025 [32]	NSCLC	CAF-derived FBLN5 impairs ferroptosis	Integrin αVβ5–Src–STAT3 → ACSL4 ↓ → ferroptosis ↓	Target FBLN5/integrin αVβ5/Src/STAT3; restore ACSL4/ferroptosis
Guo et al., 2023 [33]	Breast	CAF-derived IL-6 activates tumor STAT3	IL-6–JAK/STAT3	Anti-IL-6/IL-6R; STAT3 inhibitors
Huang et al., 2021 [34]	NPC	Senescent CAF SASP with IL-8 promotes survival	IL-8–NF-κB	Block IL-8/CXCR; inhibit NF-κB
Zhang et al., 2023 [35]	NSCLC	CAF-driven glycolysis supports tumor DDR	Glycolysis (HK2) → ATM/BRCA1 (DDR)	Inhibit glycolysis + RT
Chen et al., 2020 [36]	CRC	CAF exosomal miR-590-3p suppresses CLCA4	miR-590-3p–CLCA4–PI3K/AKT	Anti-miR-590-3p; restore CLCA4; block exosomes

### 3.3. Immune Components: TIL–PDO Co-Cultures

Tumor-infiltrating lymphocytes (TILs) critically shape solid tumor immunology, with their abundance, composition, and spatial organization correlating strongly with prognosis across cancer types [37,38,39,40,41]. Co-culture platforms integrating stromal/immune compartments with PDOs recapitulate TME crosstalk while retaining native morphology and immune constituents [42,43]. For instance, melanoma PDOs preserve tumor heterogeneity and diverse immune populations—including T cells and myeloid subsets—enabling immunotherapy screening [42]. Mechanistic studies using autologous TIL-PDO systems reveal that PD-1 blockade activates tumor innate lymphoid cells (TILCs) to sustain antitumor T-cell responses [44,45]. In CRC, Fusobacterium nucleatum enhances PD-L1 blockade efficacy by activating STING/NF-κB pathways, upregulating PD-L1, and recruiting IFN-γ^+^ CD8^+^ TILs—validated in PDO co-cultures via suppressed proliferation and induced apoptosis (Figure 1C) [46].

Collectively, TIL-PDO platforms decode mechanisms of immune checkpoint blockade (ICB), identify microbiota–immune conditioning of therapeutic response, and provide clinically translatable insights.

### 3.4. Vascularization and Organoid-on-a-Chip(OoC) Platforms

Achieving perfusable, hierarchically organized, and long-term imageable vascular networks is pivotal for advancing tumor organoids from structural mimicry to functional readouts and translational evaluation. The vascular niche not only supplies oxygen and nutrients and establishes mechanical flow fields, but also delivers angiocrine signals that regulate tumor stemness, invasion, and therapeutic response [47]. Persistent limitations in conventional models—namely inadequate vascular network fidelity, incomplete biological realism, and constraints in chip architecture—have consequently catalyzed the rapid development of vascularized OoC systems and diverse vascularization strategies.

To more faithfully recapitulate the perivascular niche and its role in metastasis, Du et al. developed a personalized vascularized OoC. The platform integrates PDOs with self-assembled, perfusable, hierarchical microvascular networks in a multichannel device containing a microvascular channel, an open-top organoid chamber, and a nutrient channel, enabling parallel assays and long-term, high-resolution visualization of tumor–vascular dynamics [23]. In osteosarcoma PDOs, the extent of angiogenesis and directed organoid migration toward vessels aligned with patients’ metastatic outcomes, providing a patient-proximal readout of metastatic propensity. Mechanistically, microvessel co-culture activated Notch signaling to drive migration, while the VEGFR2 inhibitor apatinib selectively suppressed tumor-induced sprouting angiogenesis and reversed microvessel-induced transcriptional changes, with minimal effects on mature vessels (Figure 1D) Collectively, OoC offers a physiologically relevant, personalized testbed for dissecting metastasis biology, stratifying metastatic risk, and evaluating anti-angiogenic therapies.

## 4. Predicting Radiotherapy Response with PDOs

The clinical impact of targeted and immunotherapies is often limited by primary or acquired resistance, and conventional cell lines insufficiently capture the diversity of therapeutic targets—particularly for rare cancers lacking robust preclinical models [48]. In this context, PDOs show strong concordance with clinical outcomes and have been used to predict chemotherapy and/or radiotherapy efficacy across metastatic gastrointestinal malignancies [49,50], lung cancer [51], pancreatic cancer [52,53], and other tumor types (Table 3).

PDOs provide a powerful platform for predicting radiotherapy response. Hsu et al. showed that PDOs faithfully recapitulate intrinsic tumor radiosensitivity: clonogenic survival curves fitted by the single-hit multi-target (SHMT) model yield quantitative D_0_ values that track radiation response. Whereas normal colon and adenoma PDOs tend to be radioresistant, a subset of CRC PDOs is markedly radiosensitive, and is frequently associated with homologous recombination defects (mutational signature 3). In rectal cancer, PDO D_0_ values inversely correlated with clinical response to neoadjuvant chemoradiotherapy (nCRT), with radiosensitive PDOs aligning with complete or major regression [54]. Complementing these findings, Mu et al. reported that radioresistant BRAF-mutant CRC organoids mirrored postoperative tumor persistence, while in vitro synergy between 5-FU and radiation paralleled clinical chemoradiation efficacy—together establishing PDOs as a predictive tool for tailoring radiation-based strategies in CRC [55].

**Table 3:** Predicting radiotherapy response with PDOs.

Study (Year)	Cancer Type	PDO RT Assay/Readout	Linked Clinical Endpoint	Predictive Linkage (Concise)
Yao et al. (2020) [56]	LARC	Ex vivo CRT exposure; viability/colony formation; DNA damage/apoptosis readouts	Tumor regression grade (TRG, Dworak), clinical complete response (cCR) after neoadjuvant CRT	PDO sensitivity stratified clinical responders vs. non-responders; organoid readouts mirrored TRG/cCR grouping.
Ganesh et al. (2019) [57]	CRC	Dose response to RT ± chemo; survival/repair metrics	Tumor downsizing/response to preoperative therapy (endoscopic diameter shrinkage; near/complete clinical response strata)	Concordant ranking between PDO radioresponse and clinical shrinkage strata; proof-of-concept for individualized CRT planning.
Issing et al. (2025) [58]	HNSCC	Clonogenic survival after RT; growth rate metrics (GR/GRinf)	Local control/recurrence from linked longitudinal data	GRinf-based stratification associated with recurrence risk; poor radiosensitivity linked to recurrence, favorable linked to ≥2-year non-recurrence.
Li et al. (2025) [59]	HNSCC	0–8 Gy viability dose response (CellTiter-Glo); AUC/curve	Case-level RT response	Case-level concordance observed; small sample size—predictive utility requires larger validation.
Mu et al. (2025) [55]	CRC	PDO radioresponse stratified by genotype (e.g., BRAFV600E)	External rectal cohorts: TRG and survival after CRT	BRAFV600E linked to PDO radioresistance; RAF inhibitor + CRT improved PDO response; clinical cohorts confirmed worse TRG and survival in BRAFV600E.

## 5. Mechanisms of Radioresistance Elucidated by PDOs

RT deploys high-energy ionizing radiation (IR) to kill tumor cells primarily by compromising DNA integrity and cellular homeostasis. Its cytotoxicity arises through convergent mechanisms: induction of DNA double-strand breaks (DSBs), generation of reactive oxygen species (ROS), disruption of cell-cycle progression, and engagement of regulated cell-death pathways such as apoptosis, autophagy, and necrosis [1]. In parallel, IR can elicit immunogenic cell death (ICD), releasing danger-associated molecular patterns (DAMPs)—notably calreticulin, ATP, and HMGB1—to prime antitumor immunity [60].

Despite these effects, radioresistance remains a major barrier to durable control. Five biological programs consistently underpin resistance: (i) augmented DNA damage response (DDR) and repair capacity; (ii) enrichment and plasticity of CSCs; (iii) hypoxia-driven metabolic and redox adaptation; (iv) immune evasion with remodeling of the tumor immune microenvironment (TIME); and (v) radiation-induced senescence. Together, these processes enhance survival, enable recovery from radiation-induced stress, and foster relapse [60]. The following subsections synthesize PDO-based insights into DDR, CSC biology, hypoxia/oxidative stress, and immune remodeling, and outline opportunities for therapeutic intervention.

### 5.1. DNA Damage Response and Repair Pathways

The capacity of cancer cells to detect, signal, and repair such damage—the DNA damage response (DDR)—is a principal determinant of radiosensitivity. Hyperactivation of DDR pathways enables efficient repair of radiation-induced lesions, thereby promoting survival and fostering treatment resistance. Core DDR nodes include the kinases ATM/ATR and DNA-PKcs, together with effectors such as BRCA1/2 and RAD51 that coordinate non-homologous end joining (NHEJ) and homologous recombination (HR) to resolve DSBs [61,62,63,64,65]. Pharmacologic inhibition of these pathways—using ATM, ATR, DNA-PK, or PARP inhibitors—can impede repair, increase vulnerability to DNA damage, and ultimately enhance cell death.

PDOs provide a physiologically relevant ex vivo system to interrogate DDR mechanisms and evaluate radiosensitization strategies in gastrointestinal cancers. In gastric cancer, PDO studies showed that targeting MCM6—an oncogenic transcriptional target of YAP—suppresses ATR–Chk1-mediated DDR signaling, thereby sensitizing tumors to 5-FU and irradiation [66]. In CRC, PDOs identified a radioresistant PROX1^+^ stem/progenitor subpopulation with elevated expression of DDR genes, including components of NHEJ and base excision repair (BER). Following irradiation, PROX1^+^ cells expand, whereas combining RT with the NHEJ inhibitor SCR7 curtails their survival; conversely, PROX1-deficient organoids display impaired viability after irradiation, underscoring DDR’s role in maintaining resilience [67]. Transcriptomic profiling of rectal cancer organoids (e.g., HUB005, HUB183) further revealed that radioresistant subtypes upregulate numerous DNA repair programs and antioxidant metabolism—particularly the glutamate-cysteine ligase catalytic subunit (GCLC), the rate-limiting enzyme in glutathione synthesis—whereas radiosensitive organoids preferentially activate pro-apoptotic pathways. CRISPR–Cas9 knockout of GCLC markedly augments radiation response, validating GCLC as a radiosensitization target [68]. Other studies targeting DDR pathways have also been conducted using PDOs [69,70,71,72]. Collectively, these findings highlight PDOs as powerful platforms for dissecting DDR-dependent resistance and guiding the development of effective radiosensitizers.

### 5.2. Cancer Stem Cells

CSCs constitute a functionally distinct tumor subpopulation defined by self-renewal, differentiation plasticity, and strong tumor-initiating capacity [73]. They are key drivers of recurrence, metastasis, and resistance to chemo and radiotherapy [74,75]. PDOs offer a physiologically relevant, heterogeneous ex vivo platform for interrogating CSC-targeted strategies. Mechanistically, CSCs display augmented DNA repair—particularly via HR and NHEJ—together with efficient ROS scavenging, apoptosis resistance, and a quiescent cell cycle state that collectively confer radioresistance. Core developmental pathways (Wnt/β-catenin, Notch, Hedgehog, Hippo) are typically hyperactivated, sustaining stemness under genotoxic stress [76]. Notably, radiation can further exacerbate resistance by inducing dedifferentiation of non-CSCs into CSC-like cells through epigenetic reprogramming and inflammatory cytokine signaling, thereby amplifying intratumoral heterogeneity.

To counter CSC-driven radioresistance, multiple therapeutic avenues have been explored [77,78], including pharmacologic blockade of stemness pathways (e.g., PF-03084014 and MK-0752 for Notch [79]; vismodegib for Hedgehog [80]), metabolic reprogramming [81], and epigenetic modulation (HDAC and DNMT inhibitors). These approaches impair self-renewal, attenuate DNA repair capacity, and enhance radiosensitivity. In colorectal cancer, PDOs preserve stemness features and markers (CD44, CD133, LGR5, ALDH1A1), enabling functional evaluation of anti-CSC regimens. Radiosensitization strategies combining HDAC or Wnt inhibitors with RT reduce post-irradiation regrowth, in part by downregulating CSC markers and diminishing survival of stem-like subpopulations [82].

Organoid models also reveal stem cell-driven differences in radiosensitivity across tissues: small intestinal organoids are more radiation-vulnerable than colonic organoids, consistent with the clinical prevalence of radiation enteritis [83]. Furthermore, small-molecule or antibody inhibitors targeting Wnt, Notch, or the c-KIT/SLUG axis—such as bufalin—can suppress CSC traits, induce apoptosis, and curb organoid growth. Crucially, PDOs capture interpatient variability in drug response, supporting their use in developing personalized, CSC-directed radiosensitization strategies [84].

### 5.3. Hypoxia, Redox Homeostasis, and Metabolic Adaptation

Tumor hypoxia—a hallmark of many solid malignancies—is one driver of radioresistance [85]. According to the oxygen fixation hypothesis, sufficient molecular oxygen is required to “fix” radiation-induced DNA lesions via ROS. In hypoxic niches, limited oxygen availability dampens ROS generation and reduces DSBs, thereby attenuating radiation efficacy. Beyond limiting ROS-mediated injury, hypoxic tumors reinforce intrinsic defenses by upregulating antioxidant systems—including glutathione peroxidase (GSH-Px), superoxide dismutase (SOD), and catalase—which further neutralize ROS and protect against irradiation-induced oxidative stress [86].

Glioblastoma multiforme (GBM) exemplifies hypoxia-driven radioresistance [87]. Hubert et al. established a three-dimensional, patient-derived GBM organoid platform that spontaneously forms oxygen gradients closely mirroring the in vivo microenvironment. As organoids expand to 3–4 mm in diameter—exceeding the ~200 μm oxygen diffusion limit—they develop an oxygenated periphery and a hypoxic core. Spatial mapping using the hypoxia marker CA-IX delineated these regions, and the core was enriched for a quiescent CSC subpopulation (SOX2-positive, Ki-67-low), recapitulating the biphasic distribution of perivascular versus distal hypoxic niches observed in native tumors. Functionally, following 3 Gy irradiation, CSCs within the hypoxic core exhibited minimal apoptosis (cleaved caspase-3–negative), whereas non-stem cells in oxygenated areas underwent pronounced apoptosis. These findings validate hypoxia as a central determinant of CSC-mediated radioresistance and underscore the unique ability of organoids to preserve spatial heterogeneity (e.g., regional hypoxia, stem-cell hierarchy). Consequently, this platform provides a physiologically relevant setting for screening hypoxia-targeted radiosensitization strategies [88].

### 5.4. Immune Evasion and TIME Remodeling

Combining RT with immunotherapy (IT) can synergistically enhance antitumor effi-cacy by reshaping the TIME. RT exerts a dual influence: it can potentiate antitumor immunity yet also foster immunosuppressive niches that sustain radioresistance [89].

On the immunostimulatory side, RT induces immunogenic cell death (ICD), marked by calreticulin exposure and ATP/HMGB1 release, thereby promoting dendritic cell (DC) antigen presentation and adaptive immune activation [90,91]. Cytosolic DNA generated by RT activates cGAS–STING signaling, driving type I interferon (IFN) production and DC maturation [92,93]. At higher doses, however, RT can upregulate the exonuclease TREX1, which degrades cytosolic DNA, dampens STING signaling, and blunts immune cell recruitment—illustrating a dose- and context-dependent balance [94]. STING activity is further required for the induction of chemokines and effectors that recruit and sustain T-cell responses.

Organoid models are well suited to interrogate these pathways. In colorectal cancer organoids, pharmacologic inhibition of SIRT2 (AGK2) promotes MLH1 degradation, activates cGAS–STING, elevates type I IFNs and chemokines, and enhances anti–PD-1 efficacy, including in pMMR settings that are typically immunotherapy-refractory [95]. In patient-derived melanoma organoids, the TTK inhibitor OSU13 induces DNA damage and micronuclei formation, triggering robust secretion of CCL5 and CXCL10 and reducing tumor viability. Together, these studies illustrate how organoids can evaluate both direct antitumor effects and the capacity of agents to engage innate immune sensing and remodel the tumor–immune interface [96].

Conversely, RT can drive immunosuppressive remodeling. By inducing a senescence-associated secretory phenotype (SASP) and elevating cytokines such as TGF-β and CCL2, RT recruits M2-like tumor-associated macrophages (TAMs) and myeloid-derived suppressor cells (MDSCs) [97]. RT-modulated chemokines (e.g., CCL2, CXCL1, CSF1) further facilitate myeloid infiltration. These cells secrete IL-10, TGF-β, and Arg1, suppressing CD8^+^ T-cell function and promoting epithelial–mesenchymal transition (EMT) and progression [23]. Regulatory T cells (Tregs) are also recruited via CCL17/CCL22 and suppress effector T cells through IL-10, IL-35, and adenosine [98].

Organoid–immune co-culture systems enable individualized modeling of TIME dynamics under RT and checkpoint blockade. In melanoma, PDO–TIL co-cultures treated with anti-PD-1 show viability changes concordant with clinical responses [99]; in lung cancer organoids, PD-1 blockade reverses T-cell exhaustion and restores effector phenotypes, mirroring in vivo behavior [100]. Overall, organoid platforms bridge mechanistic inquiry and patient-tailored immunotherapy design, particularly for evaluating novel immunomodulators and rational RT–IT combinations.

### 5.5. Radiation-Induced Senescence in Cancers

RT also induces therapy-induced senescence (TIS), characterized by persistent SA-β-Gal activity and SASP. TIS rewires transcriptional/epigenetic programs and reprograms the tumor microenvironment, thereby seeding post-radiation recurrence and resistance [101,102]. In glioblastoma, an SA-β-Gal^+^ subpopulation emerging after irradiation upregulates tissue factor F3 (CD142); F3 supports clonal regrowth and, via coagulation and intracellular signaling, drives mesenchymal-like transdifferentiation, enhances chemokine secretion, activates tumor-associated macrophages, and remodels extracellular matrix—collectively fostering a pro-malignant senescence network and radioresistance [103]. Radioresistant states also couple to ferroptosis evasion: senescence-linked IFI16 is upregulated and, through transcription factors such as JUND, induces HMOX1 to reduce lipid peroxidation and ROS/Fe^2+^, suppressing ferroptosis. Genetic or pharmacologic inhibition of IFI16/HMOX1 reverses these effects and restores radiosensitivity, highlighting a tractable “senescence–ferroptosis” axis [104].

PDOs are pivotal for elucidating these mechanisms because they preserve native architecture and intratumoral heterogeneity, enabling near-physiologic observation of radiation-induced senescence and its upstream triggers. In glioblastoma studies, parallel validation across patient tissue slices and organoids confirms irradiation-induced increases in SA-β-Gal, with F3 induction reproduced in tumor spheres, organoids, and patient sections—providing cross-model evidence for the TIS–F3–microenvironment remodeling–resistance cascade [103]. PDOs also enable functional dissection of senescence–immunity crosstalk: in gastric cancer, AURK inhibitor-treated organoids exhibit canonical senescence (enhanced SA-β-Gal, multinucleation) and secrete high CCL2; in PDO–macrophage co-culture, the CCL2–CCR2 axis recruits and polarizes M2 macrophages, dampening innate tumor cell clearance. This experimentally tractable framework transfers to RT-induced senescence, allowing PDOs to both visualize TIS and reconstruct its immune consequences, while cross-validating with in vivo data [105]. Consequently, PDO platforms are well suited to screen and optimize rational radiosensitizing combinations—such as senolytics, anticoagulation targeting the F3 axis, myeloid reprogramming, and ferroptosis-enhancing strategies—under patient-proximal conditions [103].

### 5.6. Epigenetic Regulation of Radioresistance

Radioresistance is not solely a consequence of genetic alterations; it is increasingly understood as an epigenetically driven phenotype shaped by DNA methylation, histone modifications, and non-coding RNA–mediated transcriptional programs. Ionizing radiation itself induces broad chromatin remodeling, influencing DDR pathway choice, checkpoint activation, and cell-fate decisions. Recent work demonstrates that these epigenetic layers are essential determinants of whether irradiated cells undergo repair, senescence, or apoptosis [106]. A major advantage of PDOs is their ability to retain patient-specific epigenomic states faithfully. This makes them highly suitable for dissecting epigenetically encoded radioresponse programs. For example, in renal cancer organoid models, epigenetic silencing of KAT2B through promoter hypermethylation was shown to drive metabolic reprogramming and tumor progression; restoration of KAT2B or inhibition of its downstream target of FASN-suppressed organoid growth [107]. Although not a radiotherapy study, this work exemplifies how PDOs can reveal clinically actionable epigenetic vulnerabilities, an approach readily adaptable to radiosensitization research.

Epigenetic dysregulation also plays a role in RT-induced normal tissue toxicity. Human cerebral organoids irradiated in vitro recapitulate the widespread DNA hypomethylation and disruption of neurodevelopmental and inflammatory programs observed in irradiated brain tissue [108]. This demonstrates the value of organoid platforms not only for tumor radioresistance studies but also for understanding—and potentially mitigating—late radiation toxicities.

## 6. PDO-Enabled Discovery of Radiosensitizers

### 6.1. Dual-Organoid Strategy for Efficacy–Toxicity Profiling

PDOs have transformed preclinical modeling by preserving the three-dimensional architecture, molecular heterogeneity, and treatment responsiveness of primary tumors. Building on this foundation, paired organoid systems—comprising tumor-derived organoids (PDTOs) and matched normal organoids from the same patient—offer a more comprehensive and clinically relevant framework for therapeutic evaluation. By enabling side-by-side testing under identical conditions, this dual-organoid approach permits simultaneous assessment of tumor-selective radiosensitization and normal tissue toxicity, directly addressing a central challenge in radiotherapy development: optimizing therapeutic efficacy while minimizing collateral damage.

In most dual-organoid studies, matched “normal” PDOs are derived from non-malignant epithelium of the same organ as the tumor. For example, Park et al. established paired CRC PDOs and normal colonic PDOs from surgical and endoscopic specimens of colorectal cancer patients. Normal PDOs were generated from adjacent non-tumor mucosa and expanded as epithelial crypt-derived organoids in Matrigel with Wnt agonists, whereas CRC PDOs were selectively maintained in Wnt-free medium, thereby enriching for malignant clones. Using this paired system, butyrate enhanced the effect of fractionated γ-irradiation in CRC PDOs, while sparing or even supporting the regenerative capacity of normal PDOs under the same treatment conditions [109].

Similarly, Nag et al. employed human colonic organoids derived from matched malignant and non-malignant tissues to interrogate auranofin as a combined radioprotector and radiosensitizer [110]. In this setting, auranofin pretreatment protected non-malignant intestinal organoids and mouse crypt epithelium from radiation-induced injury, yet concurrently sensitized malignant colonic organoids and tumors to irradiation. These platforms illustrate that most “normal” PDOs currently used for dual-organoid efficacy–toxicity profiling are purely epithelial cultures embedded in basement membrane extract, without routine co-culture of stromal fibroblasts, endothelial cells, or immune populations. While this design provides a controlled epithelial readout of normal tissue toxicity, it underestimates the contribution of stromal and immune compartments to organ-level toxicity, highlighting an opportunity to extend paired PDO systems towards more complex, TME-informed co-culture configurations in future radiotherapy studies.

### 6.2. PDO-Guided Design of Nanoradiosensitizers

Nanoparticles can potentiate radiotherapy by amplifying DDR and ROS generation, while also serving as vehicles for targeted drug delivery, oxygen modulation, and multimodal imaging [111,112,113,114]. PDOs have become indispensable for developing and optimizing nanoradiosensitizers because they preserve patient-specific architecture, molecular profiles, and therapeutic responses. Crucially, integrating paired PDTOs with matched normal organoids from the same patient enables concurrent readouts of tumor-selective efficacy and off-target toxicity [115], thereby sharpening estimates of the therapeutic index and accelerating translation.

Recent work illustrates how PDOs inform the rational design of multifunctional nanoplatforms. Using colorectal cancer PDOs, Gu et al. showed that a vitamin K2-based nanobooster (VK-OVA@HMP) augments PDT-induced immunity by promoting immunogenic cell death and dendritic cell maturation, while matched normal organoids confirmed minimal toxicity [23]. Similarly, Ye et al. validated a near infrared-responsive platform (IT-4F NPs) that couples PDT with photothermal effects to trigger robust PANoptosis, a form of inflammatory regulated cell death that integrates pyroptosis, apoptosis, and necroptosis, and potent immune activation; the antitumor activity was confined to cancer organoids without impairing paired normal organoids, underscoring selective action [116]. Extending this paradigm to therapy resistance, Chen et al. leveraged paired PDOs from cisplatin-resistant colorectal tumors to evaluate a hypoxia-amplifying, DNA repair-inhibiting nanomedicine (HYDRI NM) [117]. The platform revealed enhanced intracellular hypoxia and selective DDR inhibition in drug-resistant cancer organoids, with negligible cytotoxicity in normal counterparts.

Collectively, these studies demonstrate that paired PDO systems provide a robust, clinically relevant framework to (i) identify patient-specific vulnerabilities (e.g., hypoxia, DDR dependencies), (ii) deconvolute mechanisms of action and selectivity, and (iii) prioritize nanoradiosensitizers with favorable therapeutic indices—thereby accelerating discovery, biomarker development, and personalized translation.

### 6.3. High-Throughput Organoid Screening for Radiosensitization

High-throughput screening using patient-derived organoids (PDOs) enables systematic identification of radiosensitizers in a clinically relevant context. A 384-well PDO assay was used to test large drug libraries under irradiated and non-irradiated conditions, with radiosensitization quantified by ΔAUC. Across multiple PDOs, RAS–MAPK inhibitors—particularly MEK inhibitors—emerged as consistent radiosensitizing candidates. Multi-dose matrix assays further revealed strong synergy between MEK inhibition, PARP inhibition, and radiation [118]. These findings, validated in PDOs, highlight how organoid-based high-throughput platforms can uncover pathway-level vulnerabilities and guide rational design of effective drug–radiotherapy combinations.

## 7. Conclusions

PDOs are emerging as a practical bridge between radiobiology and clinical decision-making. By preserving patient-specific genetics, 3D architecture, and key aspects of intratumoral heterogeneity, they can help interrogate drivers of radioresistance —including dysregulated DDR, CSC plasticity, hypoxia and redox imbalance, TIME remodeling, and radiation-induced senescence—and provide patient contexts for exploring rational radiosensitizer combinations. When paired with matched normal organoids, these platforms may also support side-by-side efficacy–toxicity assessment, which is helpful for optimizing therapeutic index and prioritizing candidates for translational testing.

Despite these strengths, important limitations remain. PDO establishment efficiency is highly tumor-type–dependent. While colorectal, gastric, and pancreatic cancers generally show high success rates, deriving stable PDOs from certain malignancies—such as primary prostate cancers, some sarcomas, and heavily pretreated or fibrotic tumors—remains technically challenging. These constraints may introduce selection bias toward more proliferative or treatment-resistant clones, limiting the representativeness of PDO cohorts. Therefore, rigorous validation of PDO fidelity is essential. Standard approaches typically include side-by-side comparisons of PDOs and their parental tumors at the histological level, immunophenotypic profiling to confirm lineage identity, genomic characterization to verify conservation of driver mutations and pathway alterations, and—where available—correlation with clinical radiotherapy responses. Such multilayered benchmarking is critical for establishing that PDOs faithfully model the tumor type from which they are derived.

Another key consideration is the impact of serial passaging. Although major driver mutations often remain stable across early passages, cumulative passaging can modify clonal composition, stem/progenitor fractions, proliferative kinetics, and ultimately radioresponse phenotypes. Inter-passage variability has been reported in both morphological features and treatment sensitivity, underscoring the importance of using early-passage PDOs for predictive modeling, documenting passage numbers in all experiments, and replicating key findings across several passage windows. Systematic future studies quantifying long-term genomic and functional drift will be essential for defining the “predictive window” during which PDOs retain maximal biological relevance.

Emerging standardization efforts—such as those led by the EORTC and NIH PDO consortia—are now establishing unified protocols and quality benchmarks, which will be essential for ensuring reproducibility and advancing PDO-based radiotherapy models toward clinical validation. Future work should prioritize the development of standardized PDO culture and irradiation workflows capable of modeling clinically relevant fractionation schemes. By integrating advanced irradiation platforms (including micro-field and ultra-high-precision dose delivery), longitudinal live imaging, and computational modeling, PDO systems may more faithfully recapitulate the classical “4R” radiobiological processes—repair, repopulation, reoxygenation, and redistribution—thereby greatly enhancing their value in predicting individualized radiotherapy responses and enabling the discovery of novel radiosensitizers.

Looking ahead, the convergence of PDOs with high-content imaging, single-cell and spatial omics, CRISPR functional genomics, biomimetic ECM engineering and advanced irradiation platforms—including FLASH, proton, and carbon-ion radiotherapy—will accelerate the discovery of actionable resistance mechanisms and clinically tractable radiosensitizers. The integration of machine learning-based response modeling and larger biobank-linked PDO cohorts will further support individualized radiotherapy stratification. Continued refinement of culture conditions, stromal co-culture systems, and passage-aware quality control frameworks will be essential for unlocking the full potential of PDOs as next-generation tools for mechanistic studies, therapeutic screening, and personalized clinical decision-making in radiation oncology.

## Figures and Tables

**Table 1:** Comparison of PDOs and common preclinical model systems.

Feature	2D Cell Lines	PDOs	PDXs
Structure	Flat monolayer	3D tissue-like	Patient tumor in mouse
TME components	None	Minimal	Mouse stroma
Heterogeneity	Very low	High	High
Scalability/Throughput	Very high	Moderate	Low
Cost/Time	Low/Fast	Moderate/Medium	High/Slow
Relevance to human RT	Low	High	High

## Data Availability

No new data were created or analyzed in this study. Data sharing is not applicable to this article.

## Abbreviations

The following abbreviations are used in this manuscript:

BED	Biologically effective dose
CAF	Cancer-associated fibroblast
CIRT	Carbon-ion radiotherapy
CRT	Chemoradiotherapy
cCR	Clinical complete response
DDR	DNA damage response
DSB	Double-strand break
ECM	Extracellular matrix
ESCC	Esophageal Squamous Cell Carcinoma
FLASH	Ultra-high dose rate radiotherapy
GBM	Glioblastoma
GR	Growth rate
GRinf	Growth rate at infinite time
HNSCC	Head and neck squamous cell carcinoma
LARC	Local advanced rectal cancer
LET	Linear energy transfer
NPC	Nasopharyngeal carcinoma
NSCLC	Non-small-cell lung cancer
OoC	Organoid-on-a-chip
PBT	Proton beam therapy
PD-L1	Programmed death-ligand 1
PDO	Patient-derived organoid
PDX	Patient-derived xenograft
ROS	Reactive oxygen species
RT	Radiotherapy
SA-β-Gal	Senescence-associated β-galactosidase
SASP	Senescence-associated secretory phenotype
SF2	Surviving fraction at 2 Gy
TAM	Tumor-associated macrophage
TNBC	Triple-negative Breast Cancer
TIL	Tumor-infiltrating lymphocyte
TIS	Therapy-induced senescence
TME	Tumor microenvironment
TF	Tissue factor (gene F3)
TRG	Tumor Regression Grade
γH2AX	Phosphorylated H2AX (Ser139), a DSB marker
D_0_	Mean lethal dose (single-hit model)

## References

[1] An L., Li M., Jia Q. (2023). Mechanisms of radiotherapy resistance and radiosensitization strategies for esophageal squamous cell carcinoma. Mol. Cancer.

[2] Ma X., Yang S., Jiang H., Wang Y., Xiang Z. (2021). Transcriptomic analysis of tumor tissues and organoids reveals the crucial genes regulating the proliferation of lung adenocarcinoma. J. Transl. Med..

[3] Niklinska-Schirtz B.J., Venkateswaran S., Anbazhagan M., Kolachala V.L., Prince J., Dodd A., Chinnadurai R., Gibson G., Denson L.A., Cutler D.J. (2021). Ileal Derived Organoids From Crohn’s Disease Patients Show Unique Transcriptomic and Secretomic Signatures. Cell. Mol. Gastroenterol. Hepatol..

[4] Gong L., Zhang Y., Liu C., Zhang M., Han S. (2021). Application of Radiosensitizers in Cancer Radiotherapy. Int. J. Nanomed..

[5] Sato T., Vries R.G., Snippert H.J., Van De Wetering M., Barker N., Stange D.E., Van Es J.H., Abo A., Kujala P., Peters P.J. (2009). Single Lgr5 Stem Cells Build Crypt-Villus Structures in Vitro without a Mesenchymal Niche. Nature.

[6] Sato T., Stange D.E., Ferrante M., Vries R.G.J., Van Es J.H., Van Den Brink S., Van Houdt W.J., Pronk A., Van Gorp J., Siersema P.D. (2011). Long-term Expansion of Epithelial Organoids From Human Colon, Adenoma, Adenocarcinoma, and Barrett’s Epithelium. Gastroenterology.

[7] Van De Wetering M., Francies H.E., Francis J.M., Bounova G., Iorio F., Pronk A., Van Houdt W., Van Gorp J., Taylor-Weiner A., Kester L. (2015). Prospective derivation of a living organoid biobank of colorectal cancer patients. Cell.

[8] Zu M., Hao X., Ning J., Zhou X., Gong Y., Lang Y., Xu W., Zhang J., Ding S. (2023). Patient-derived organoid culture of gastric cancer for disease modeling and drug sensitivity testing. Biomed. Pharmacother..

[9] Lian G., Malagola E., Wei C., Shi Q., Zhao J., Hata M., Kobayashi H., Ochiai Y., Zheng B., Zhi X. (2024). p53 mutation biases squamocolumnar junction progenitor cells towards dysplasia rather than metaplasia in Barrett’s oesophagus. Gut.

[10] Madorsky Rowdo F.P., Xiao G., Khramtsova G.F., Nguyen J., Martini R., Stonaker B., Boateng R., Oppong J.K., Adjei E.K., Awuah B. (2024). Patient-derived tumor organoids with p53 mutations, and not wild-type p53, are sensitive to synergistic combination PARP inhibitor treatment. Cancer Lett..

[11] Raaijmakers K.T.P.M., Adema G.J., Bussink J., Ansems M. (2024). Cancer-associated fibroblasts, tumor and radiotherapy: Interactions in the tumor micro-environment. J. Exp. Clin. Cancer Res..

[12] de Visser K.E., Joyce J.A. (2023). The evolving tumor microenvironment: From cancer initiation to metastatic outgrowth. Cancer Cell.

[13] Sahai E., Astsaturov I., Cukierman E., DeNardo D.G., Egeblad M., Evans R.M., Fearon D., Greten F.R., Hingorani S.R., Hunter T. (2020). A framework for advancing our understanding of cancer-associated fibroblasts. Nat. Rev. Cancer.

[14] Palucka A.K., Coussens L.M. (2016). The basis of oncoimmunology. Cell.

[15] Fridman W.H., Zitvogel L., Sautès-Fridman C., Kroemer G. (2017). The immune contexture in cancer prognosis and treatment. Nat. Rev. Clin. Oncol..

[16] Schreiber R.D., Old L.J., Smyth M.J. (2011). Cancer Immunoediting: Integrating Immunity’s Roles in Cancer Suppression and Promotion. Science.

[17] Garreta E., Moya-Rull D., Marco A., Amato G., Ullate-Agote A., Tarantino C., Gallo M., Esporrín-Ubieto D., Centeno A., Vilas-Zornoza A. (2024). Natural Hydrogels Support Kidney Organoid Generation and Promote In Vitro Angiogenesis. Adv. Mater..

[18] Mosquera M.J., Kim S., Bareja R., Fang Z., Cai S., Pan H., Asad M., Martin M.L., Sigouros M., Rowdo F.M. (2021). Extracellular Matrix in Synthetic Hydrogel-Based Prostate Cancer Organoids Regulate Therapeutic Response to EZH2 and DRD2 Inhibitors. Adv. Mater..

[19] Lu P., Weaver V.M., Werb Z. (2012). The extracellular matrix: A dynamic niche in cancer progression. J. Cell Biol..

[20] Aebersold R., Mann M. (2016). Mass-spectrometric exploration of proteome structure and function. Nature.

[21] Biondani G., Zeeberg K., Greco M.R., Cannone S., Dando I., Pozza E.D., Mastrodonato M., Forciniti S., Casavola V., Palmieri M. (2018). Extracellular matrix composition modulates PDAC parenchymal and stem cell plasticity and behavior through the secretome. FEBS J..

[22] Xu R., Zhou X., Wang S., Trinkle C. (2020). Tumor organoid models in precision medicine and investigating cancer-stromal interactions. Pharmacol. Ther..

[23] Du Y., Wang Y., Bao Q., Xu X., Xu C., Wang S., Liu Q., Liu F., Zeng Y., Wang Y. (2024). Personalized Vascularized Tumor Organoid-on-a-Chip for Tumor Metastasis and Therapeutic Targeting Assessment. Adv. Mater..

[24] Sensi F., Spagnol G., Repetto O., D’ANgelo E., Biccari A., Marangio A., Guerriero A., Castillo A.A.M., Vogliardi A., Zanrè E. (2025). Patient-derived extracellular matrix from decellularized high-grade serous ovarian carcinoma tissues as a biocompatible support for organoid growth. Transl. Oncol..

[25] Naruse M., Ochiai M., Sekine S., Taniguchi H., Yoshida T., Ichikawa H., Sakamoto H., Kubo T., Matsumoto K., Ochiai A. (2021). Re-expression of REG family and DUOXs genes in CRC organoids by co-culturing with CAFs. Sci. Rep..

[26] Koikawa K., Kibe S., Suizu F., Sekino N., Kim N., Manz T.D., Pinch B.J., Akshinthala D., Verma A., Gaglia G. (2021). Targeting Pin1 renders pancreatic cancer eradicable by synergizing with immunochemotherapy. Cell.

[27] Zhang R., Liu F. (2022). Cancer-associated fibroblast-derived gene signatures predict radiotherapeutic survival in prostate cancer patients. J. Transl. Med..

[28] Ohlund D., Handly-Santana A., Biffi G., Elyada E., Almeida A.S., Ponz-Sarvise M., Corbo V., Oni T.E., Hearn S.A., Lee E.J. (2017). Distinct populations of inflammatory fibroblasts and myofibroblasts in pancreatic cancer. J. Exp. Med..

[29] Yang X., Chen X., Zhang S., Fan W., Zhong C., Liu T., Cheng G., Zhu L., Liu Q., Xi Y. (2023). Collagen 1-mediated CXCL1 secretion in tumor cells activates fibroblasts to promote radioresistance of esophageal cancer. Cell Rep..

[30] Meng J., Li Y., Wan C., Sun Y., Dai X., Huang J., Hu Y., Gao Y., Wu B., Zhang Z. (2021). Targeting senescence-like fibroblasts radiosensitizes non–small cell lung cancer and reduces radiation-induced pulmonary fibrosis. J. Clin. Investig..

[31] Xun X., Hu H., Liu Q., Su R., Ai J. (2025). CAFs exosomal circFOXO1 promotes TNBC autophagy and radioresistance via miR-27a-3p/BNIP3 axis. Sci. Rep..

[32] Zhang R., Yan W., Yuan J., Ma Y., Ren Z., Chen X., Lv J., Wu M., Yu J., Chen D. (2025). Cancer-associated fibroblast-derived fibulin-5 promotes radioresistance in non-small-cell lung cancer. Cell Rep..

[33] Guo Z., Zhang H., Fu Y., Kuang J., Zhao B., Zhang L., Lin J., Lin S., Wu D., Xie G. (2023). Cancer-associated fibroblasts induce growth and radioresistance of breast cancer cells through paracrine IL-6. Cell Death Discov..

[34] Huang W., Zhang L., Yang M., Wu X., Wang X., Huang W., Yuan L., Pan H., Wang Y., Wang Z. (2021). Cancer-associated fibroblasts promote the survival of irradiated nasopharyngeal carcinoma cells via the NF-κB pathway. J. Exp. Clin. Cancer Res..

[35] Zhang H., Zhang K., Qiu L., Yue J., Jiang H., Deng Q., Zhou R., Yin Z., Ma S., Ke Y. (2023). Cancer-associated fibroblasts facilitate DNA damage repair by promoting the glycolysis in non-small cell lung cancer. Biochim. Biophys. Acta (BBA)—Mol. Basis Dis..

[36] Chen X., Liu Y., Zhang Q., Liu B., Cheng Y., Zhang Y., Sun Y., Liu J. (2021). Exosomal miR-590-3p derived from cancer-associated fibroblasts confers radioresistance in colorectal cancer. Mol. Ther.—Nucleic Acids.

[37] Salimbeni B.T., Giudici F., Pescia C., Giachetti P.P.M.B., Scafetta R., Zagami P., Marra A., Trapani D., Esposito A., Scagnoli S. (2025). Prognostic impact of tumor-infiltrating lymphocytes in HER2+ metastatic breast cancer receiving first-line treatment. npj Breast Cancer.

[38] Moreno V., Salazar R., Gruber S. (2022). The prognostic value of TILs in stage III colon cancer must consider sidedness. Ann. Oncol..

[39] Wu S.-L., Yu X., Mao X., Jin F. (2022). Prognostic value of tumor-infiltrating lymphocytes in DCIS: A meta-analysis. BMC Cancer.

[40] Yan Q., Li S., He L., Chen N. (2024). Prognostic implications of tumor-infiltrating lymphocytes in non-small cell lung cancer: A systematic review and meta-analysis. Front. Immunol..

[41] Cai C., Zhang P., Han Y., Shen H., Zeng S. (2024). Combined tumour-infiltrating lymphocytes and microsatellite instability status as prognostic markers in colorectal cancer. Lancet Gastroenterol. Hepatol..

[42] Ou L., Liu S., Wang H., Guo Y., Guan L., Shen L., Luo R., Elder D.E., Huang A.C., Karakousis G. (2023). Patient-derived melanoma organoid models facilitate the assessment of immunotherapies. EBioMedicine.

[43] Magré L., Verstegen M.M.A., Buschow S., van der Laan L.J.W., Peppelenbosch M., Desai J. (2023). Emerging organoid-immune co-culture models for cancer research: From oncoimmunology to personalized immunotherapies. J. Immunother. Cancer.

[44] Moral J.A., Leung J., Rojas L.A., Ruan J., Zhao J., Sethna Z., Ramnarain A., Gasmi B., Gururajan M., Redmond D. (2020). ILC2s amplify PD-1 blockade by activating tissue-specific cancer immunity. Nature.

[45] Demaria O., Vivier E. (2020). Immuno-Oncology beyond TILs: Unleashing TILCs. Cancer Cell.

[46] Gao Y., Bi D., Xie R., Li M., Guo J., Liu H., Guo X., Fang J., Ding T., Zhu H. (2021). *Fusobacterium nucleatum* enhances the efficacy of PD-L1 blockade in colorectal cancer. Signal Transduct. Target. Ther..

[47] Choi J.-I., Jang S.I., Hong J., Kim C.H., Kwon S.S., Park J.S., Lim J.-B. (2021). Cancer-initiating cells in human pancreatic cancer organoids are maintained by interactions with endothelial cells. Cancer Lett..

[48] Xiang D., He A., Zhou R., Wang Y., Xiao X., Gong T., Kang W., Lin X., Wang X., Consortium P.D.O.D.S.T. (2024). Building consensus on the application of organoid-based drug sensitivity testing in cancer precision medicine and drug development. Theranostics.

[49] Vlachogiannis G., Hedayat S., Vatsiou A., Jamin Y., Fernández-Mateos J., Khan K., Lampis A., Eason K., Huntingford I., Burke R. (2018). Patient-derived organoids model treatment response of metastatic gastrointestinal cancers. Science.

[50] Hogenson T.L., Xie H., Phillips W.J., Toruner M.D., Li J.J., Horn I.P., Kennedy D.J., Almada L.L., Marks D.L., Carr R.M. (2022). Culture media composition influences patient-derived organoid ability to predict therapeutic responses in gastrointestinal cancers. J. Clin. Investig..

[51] Wang H.-M., Zhang C.-Y., Peng K.-C., Chen Z.-X., Su J.-W., Li Y.-F., Li W.-F., Gao Q.-Y., Zhang S.-L., Chen Y.-Q. (2023). Using patient-derived organoids to predict locally advanced or metastatic lung cancer tumor response: A real-world study. Cell Rep. Med..

[52] Shi X., Li Y., Yuan Q., Tang S., Guo S., Zhang Y., He J., Zhang X., Han M., Liu Z. (2022). Integrated profiling of human pancreatic cancer organoids reveals chromatin accessibility features associated with drug sensitivity. Nat. Commun..

[53] Beutel A.K., Schütte L., Scheible J., Roger E., Müller M., Perkhofer L., Kestler A.M.T.U., Kraus J.M., Kestler H.A., Barth T.F.E. (2021). A Prospective Feasibility Trial to Challenge Patient–Derived Pancreatic Cancer Organoids in Predicting Treatment Response. Cancers.

[54] Hsu K.-S., Adileh M., Martin M.L., Makarov V., Chen J., Wu C., Bodo S., Klingler S., Sauvé C.-E.G., Szeglin B.C. (2022). Colorectal Cancer Develops Inherent Radiosensitivity That Can Be Predicted Using Patient-Derived Organoids. Cancer Res..

[55] Mu P., Mo S., He X., Zhang H., Lv T., Xu R., He L., Xia F., Zhou S., Chen Y. (2025). Unveiling radiobiological traits and therapeutic responses of BRAFV600E-mutant colorectal cancer via patient-derived organoids. J. Exp. Clin. Cancer Res..

[56] Yao Y., Xu X., Yang L., Zhu J., Wan J., Shen L., Xia F., Fu G., Deng Y., Pan M. (2020). Patient-Derived Organoids Predict Chemoradiation Responses of Locally Advanced Rectal Cancer. Cell Stem Cell.

[57] Ganesh K., Wu C., O’rOurke K.P., Szeglin B.C., Zheng Y., Sauvé C.-E.G., Adileh M., Wasserman I., Marco M.R., Kim A.S. (2019). A rectal cancer organoid platform to study individual responses to chemoradiation. Nat. Med..

[58] Issing C., Menche C., Richter M.R., Mosa M.H., von der Grün J., Fleischmann M., Thoenissen P., Winkelmann R., Darvishi T., Loth A.G. (2025). Head and neck tumor organoid biobank for modelling individual responses to radiation therapy according to the TP53/HPV status. J. Exp. Clin. Cancer Res..

[59] Li W., Nishino M., Reed E., Akshinthala D., Pasha H.A., Anderson E.S., Huang L., Hebestreit H., Monti S., Gomez E.D. (2025). Head and neck tumor organoid grown under simplified media conditions model tumor biology and chemoradiation responses. Sci. Rep..

[60] Jiang K., Yin X., Zhang Q., Yin J., Tang Q., Xu M., Wu L., Shen Y., Zhou Z., Yu H. (2023). STC2 activates PRMT5 to induce radioresistance through DNA damage repair and ferroptosis pathways in esophageal squamous cell carcinoma. Redox Biol..

[61] Smith H.L., Southgate H., Tweddle D.A., Curtin N.J. (2020). DNA damage checkpoint kinases in cancer. Expert. Rev. Mol. Med..

[62] Dai L., Dai Y., Han J., Huang Y., Wang L., Huang J., Zhou Z. (2021). Structural insight into BRCA1-BARD1 complex recruitment to damaged chromatin. Mol. Cell.

[63] Prakash R., Zhang Y., Feng W., Jasin M. (2015). Homologous Recombination and Human Health: The Roles of BRCA1, BRCA2, and Associated Proteins. Cold Spring Harb. Perspect. Biol..

[64] Jazayeri A., Falck J., Lukas C., Bartek J., Smith G.C.M., Lukas J., Jackson S.P. (2006). ATM- and cell cycle-dependent regulation of ATR in response to DNA double-strand breaks. Nat. Cell Biol..

[65] Greenberg R.A. (2008). Recognition of DNA double strand breaks by the BRCA1 tumor suppressor network. Chromosoma.

[66] Wang Y., Chen H., Liu W., Yan H., Zhang Y., Cheung A.H.K., Zhang J., Chen B., Liang L., Zhou Z. (2022). MCM6 is a critical transcriptional target of YAP to promote gastric tumorigenesis and serves as a therapeutic target. Theranostics.

[67] Kallio P., Bessone C., Seyednasrollah F., Brodkin J., Lassila M., Högström J., González-Loyola A., Petrova T.V., Haglund C., Alitalo K. (2025). A Multipotent PROX1+ Tumor Stem/Progenitor Cell Population Emerges During Intestinal Tumorigenesis and Mediates Radioresistance in Colorectal Cancer. Cancer Res..

[68] Andel D., Nouwens A.J., Klaassen S., Laoukili J., Viergever B., Verheem A., Intven M.P.W., Zandvliet M., Hagendoorn J., Rinkes I.H.M.B. (2025). Rational design of alternative treatment options for radioresistant rectal cancer using patient-derived organoids. Br. J. Cancer.

[69] Martincuks A., Zhang C., Austria T., Li Y.-J., Huang R., Santiago N.L., Kohut A., Zhao Q., Borrero R.M., Shen B. (2024). Targeting PARG induces tumor cell growth inhibition and antitumor immune response by reducing phosphorylated STAT3 in ovarian cancer. J. Immunother. Cancer.

[70] Awasthi S., Dobrolecki L.E., Sallas C., Zhang X., Li Y., Khazaei S., Ghosh S., Jeter C.R., Liu J., Mills G.B. (2024). UBA1 inhibition sensitizes cancer cells to PARP inhibitors. Cell Rep. Med..

[71] Caggiano C., Petrera V., Ferri M., Pieraccioli M., Cesari E., Di Leone A., Sanchez M.A., Fabi A., Masetti R., Naro C. (2024). Transient splicing inhibition causes persistent DNA damage and chemotherapy vulnerability in triple-negative breast cancer. Cell Rep..

[72] Dreyer S.B., Upstill-Goddard R., Paulus-Hock V., Paris C., Lampraki E.-M., Dray E., Serrels B., Caligiuri G., Rebus S., Plenker D. (2021). Targeting DNA Damage Response and Replication Stress in Pancreatic Cancer. Gastroenterology.

[73] Visvader J.E., Lindeman G.J. (2008). Cancer stem cells in solid tumours: Accumulating evidence and unresolved questions. Nat. Rev. Cancer.

[74] Gillespie M.S., Ward C.M., Davies C.C. (2023). DNA Repair and Therapeutic Strategies in Cancer Stem Cells. Cancers.

[75] Olivares-Urbano M.A., Griñán-Lisón C., Marchal J.A., Núñez M.I. (2020). CSC Radioresistance: A Therapeutic Challenge to Improve Radiotherapy Effectiveness in Cancer. Cells.

[76] Clara J.A., Monge C., Yang Y., Takebe N. (2019). Targeting signalling pathways and the immune microenvironment of cancer stem cells—A clinical update. Nat. Rev. Clin. Oncol..

[77] Zhou X., Gao F., Gao W., Wang Q., Li X., Li X., Li W., Liu J., Zhou H., Luo A. (2024). Bismuth Sulfide Nanoflowers Facilitated miR339 Delivery to Overcome Stemness and Radioresistance through Ubiquitin-Specific Peptidase 8 in Esophageal Cancer. ACS Nano.

[78] Zhu M., Fan H., Deng J., Jiang K., Liao J., Zhang X., Chen Y., Yu M., Peng Z. (2023). BMI1 Silencing Liposomes Suppress Postradiotherapy Cancer Stemness against Radioresistant Hepatocellular Carcinoma. ACS Nano.

[79] Wu C.X., Xu A., Zhang C.C., Olson P., Chen L., Lee T.K., Cheung T.T., Lo C.M., Wang X.Q. (2017). Notch Inhibitor PF-03084014 Inhibits Hepatocellular Carcinoma Growth and Metastasis via Suppression of Cancer Stemness due to Reduced Activation of Notch1–Stat3. Mol. Cancer Ther..

[80] Zhong Y., Chen X., Wu S., Fang H., Hong L., Shao L., Wang L., Wu J. (2024). Deciphering colorectal cancer radioresistance and immune microenvironment: Unraveling the role of EIF5A through single-cell RNA sequencing and machine learning. Front. Immunol..

[81] Guo D., Ji X., Xie H., Ma J., Xu C., Zhou Y., Chen N., Wang H., Fan C., Song H. (2023). Targeted Reprogramming of Vitamin B_3_ Metabolism as a Nanotherapeutic Strategy towards Chemoresistant Cancers. Adv. Mater..

[82] Endo H., Kondo J., Onuma K., Ohue M., Inoue M. (2020). Small subset of Wnt-activated cells is an initiator of regrowth in colorectal cancer organoids after irradiation. Cancer Sci..

[83] Martin M.L., Adileh M., Hsu K.-S., Hua G., Lee S.G., Li C., Fuller J.D., Rotolo J.A., Bodo S., Klingler S. (2020). Organoids Reveal That Inherent Radiosensitivity of Small and Large Intestinal Stem Cells Determines Organ Sensitivity. Cancer Res..

[84] Ding L., Yang Y., Lu Q., Qu D., Chandrakesan P., Feng H., Chen H., Chen X., Liao Z., Du J. (2022). Bufalin Inhibits Tumorigenesis, Stemness, and Epithelial–Mesenchymal Transition in Colorectal Cancer through a C-Kit/Slug Signaling Axis. Int. J. Mol. Sci..

[85] Hill R.M., Fok M., Grundy G., Parsons J.L., Rocha S. (2023). The role of autophagy in hypoxia-induced radioresistance. Radiother. Oncol..

[86] Shi Z., Hu C., Zheng X., Sun C., Li Q. (2024). Feedback loop between hypoxia and energy metabolic reprogramming aggravates the radioresistance of cancer cells. Exp. Hematol. Oncol..

[87] Wei J., Zhu K., Yang Z., Zhou Y., Xia Z., Ren J., Zhao Y., Wu G., Liu C. (2023). Hypoxia-Induced Autophagy Is Involved in Radioresistance via HIF1A-Associated Beclin-1 in Glioblastoma Multiforme. Heliyon.

[88] Hubert C.G., Rivera M., Spangler L.C., Wu Q., Mack S.C., Prager B.C., Couce M., McLendon R.E., Sloan A.E., Rich J.N. (2016). A Three-Dimensional Organoid Culture System Derived from Human Glioblastomas Recapitulates the Hypoxic Gradients and Cancer Stem Cell Heterogeneity of Tumors Found In Vivo. Cancer Res..

[89] Sharabi A.B., Lim M., DeWeese T.L., Drake C.G. (2015). Radiation and checkpoint blockade immunotherapy: Radiosensitisation and potential mechanisms of synergy. Lancet Oncol..

[90] Qiu H., Shao Z., Wen X., Qu D., Liu Z., Chen Z., Zhang X., Ding X., Zhang L. (2024). HMGB1/TREM2 positive feedback loop drives the development of radioresistance and immune escape of glioblastoma by regulating TLR4/Akt signaling. J. Transl. Med..

[91] Liu S., Chen J., Li L., Ye Z., Liu J., Chen Y., Hu B., Tang J., Feng G., Li Z. (2024). Susceptibility of Mitophagy-Deficient Tumors to Ferroptosis Induction by Relieving the Suppression of Lipid Peroxidation. Adv. Sci..

[92] Weichselbaum R.R., Liang H., Deng L., Fu Y.-X. (2017). Radiotherapy and immunotherapy: A beneficial liaison?. Nat. Rev. Clin. Oncol..

[93] Diamond J.M., Vanpouille-Box C., Spada S., Rudqvist N.-P., Chapman J.R., Ueberheide B.M., Pilones K.A., Sarfraz Y., Formenti S.C., Demaria S. (2018). Exosomes Shuttle TREX1-Sensitive IFN-Stimulatory dsDNA from Irradiated Cancer Cells to DCs. Cancer Immunol. Res..

[94] Vanpouille-Box C., Alard A., Aryankalayil M.J., Sarfraz Y., Diamond J.M., Schneider R.J., Inghirami G., Coleman C.N., Formenti S.C., Demaria S. (2017). DNA exonuclease Trex1 regulates radiotherapy-induced tumour immunogenicity. Nat. Commun..

[95] Gao Q., Yang L., Ye S., Mai M., Liu Y., Jiang X., Feng X., Yang Z. (2025). Targeting SIRT2 induces MLH1 deficiency and boosts antitumor immunity in preclinical colorectal cancer models. Sci. Transl. Med..

[96] Bharti V., Kumar A., Wang Y., Roychowdhury N., Bellan D.d.L., Kassaye B.B., Watkins R., Capece M., Chung C.G., Hilinski G. (2024). TTK inhibitor OSU13 promotes immunotherapy responses by activating tumor STING. J. Clin. Investig..

[97] Guo S., Yao Y., Tang Y., Xin Z., Wu D., Ni C., Huang J., Wei Q., Zhang T. (2023). Radiation-induced tumor immune microenvironments and potential targets for combination therapy. Signal Transduct. Target. Ther..

[98] Zhang Z., Liu X., Chen D., Yu J. (2022). Radiotherapy combined with immunotherapy: The dawn of cancer treatment. Signal Transduct. Target. Ther..

[99] Votanopoulos K.I., Forsythe S., Sivakumar H., Mazzocchi A., Aleman J., Miller L., Levine E., Triozzi P., Skardal A. (2019). Model of Patient-Specific Immune-Enhanced Organoids for Immunotherapy Screening: Feasibility Study. Ann. Surg. Oncol..

[100] Takahashi N., Hoshi H., Higa A., Hiyama G., Tamura H., Ogawa M., Takagi K., Goda K., Okabe N., Muto S. (2019). An In Vitro System for Evaluating Molecular Targeted Drugs Using Lung Patient-Derived Tumor Organoids. Cells.

[101] Schoetz U., Klein D., Hess J., Shnayien S., Spoerl S., Orth M., Mutlu S., Hennel R., Sieber A., Ganswindt U. (2021). Early senescence and production of senescence-associated cytokines are major determinants of radioresistance in head-and-neck squamous cell carcinoma. Cell Death Dis..

[102] Wang B., Kohli J., Demaria M. (2020). Senescent Cells in Cancer Therapy: Friends or Foes?. Trends Cancer.

[103] Jeon H.-M., Kim J.-Y., Cho H.J., Lee W.J., Nguyen D., Kim S.S., Oh Y.T., Kim H.-J., Jung C.-W., Pinero G. (2023). Tissue factor is a critical regulator of radiation therapy-induced glioblastoma remodeling. Cancer Cell.

[104] Zhou Y., Zeng L., Cai L., Zheng W., Liu X., Xiao Y., Jin X., Bai Y., Lai M., Li H. (2025). Cellular senescence-associated gene IFI16 promotes HMOX1-dependent evasion of ferroptosis and radioresistance in glioblastoma. Nat. Commun..

[105] Yang R., Kwan W., Du Y., Yan R., Zang L., Li C., Zhu Z., Cheong I.H., Kozlakidis Z., Yu Y. (2024). Drug-induced senescence by aurora kinase inhibitors attenuates innate immune response of macrophages on gastric cancer organoids. Cancer Lett..

[106] Shishido K., Reinders A., Asuthkar S. (2022). Epigenetic regulation of radioresistance: Insights from preclinical and clinical studies. Expert. Opin. Investig. Drugs.

[107] Wang Q., Miao D., Liu R., Li M., Dong Z., Liu Y., Yang C., Yang H., Wang K., Xiong Z. (2025). Epigenetically silenced KAT2B suppresses de novo lipogenesis through destroying HDAC5/LSD1 complex assembly in renal cell carcinoma. J. Adv. Res..

[108] Millner T.O., Panday P., Xiao Y., Nicholson J.G., Boot J.R., Arpe Z., Stevens P.A., Rahman N.N., Zhang X., Mein C. (2025). Disruption of DNA methylation underpins the neuroinflammation induced by targeted CNS radiotherapy. Brain.

[109] Park M., Kwon J., Shin H.J., Moon S.M., Kim S.B., Shin U.S., Han Y.H., Kim Y. (2020). Butyrate enhances the efficacy of radiotherapy via FOXO3A in colorectal cancer patient-derived organoids. Int. J. Oncol..

[110] Nag D., Bhanja P., Riha R., Sanchez-Guerrero G., Kimler B.F., Tsue T.T., Lominska C., Saha S. (2019). Auranofin Protects Intestine against Radiation Injury by Modulating p53/p21 Pathway and Radiosensitizes Human Colon Tumor. Clin. Cancer Res..

[111] Yin M., Yuan Y., Huang Y., Liu X., Meng F., Luo L., Tian S., Liu B. (2024). Carbon–Iodine Polydiacetylene Nanofibers for Image-Guided Radiotherapy and Tumor-Microenvironment-Enhanced Radiosensitization. ACS Nano.

[112] Zhang A., Gao L. (2023). The Refined Application and Evolution of Nanotechnology in Enhancing Radiosensitivity During Radiotherapy: Transitioning from Gold Nanoparticles to Multifunctional Nanomaterials. Int. J. Nanomed..

[113] Wen X., Shao Z., Chen X., Liu H., Qiu H., Ding X., Qu D., Wang H., Wang A.Z., Zhang L. (2024). A multifunctional targeted nano-delivery system with radiosensitization and immune activation in glioblastoma. Radiat. Oncol..

[114] Sood A., Arora V., Kumari S., Sarkar A., Kumaran S.S., Chaturvedi S., Jain T.K., Agrawal G. (2021). Imaging application and radiosensitivity enhancement of pectin decorated multifunctional magnetic nanoparticles in cancer therapy. Int. J. Biol. Macromol..

[115] Gu J., Zhu R., Liang R., Wang W., Li J., Zhao S., Wu Y., Cao J., Yang S., Sun Y. (2025). Molecular simulation-aided self-adjuvanting nanoamplifier for cancer photoimmunotherapy. Theranostics.

[116] Ye Y., Zhao S., Pang E., Tang Y., Zhu P., Gao W., Diao Q., Yu J., Zeng J., Lan M. (2025). Indacenodithienothiophene-based A-D-A-type phototheranostics for immuno-phototherapy. J. Nanobiotechnol..

[117] Chen J., Wang X., Yuan Y., Chen H., Zhang L., Xiao H., Chen J., Zhao Y., Chang J., Guo W. (2021). Exploiting the acquired vulnerability of cisplatin-resistant tumors with a hypoxia-amplifying DNA repair–inhibiting (HYDRI) nanomedicine. Sci. Adv..

[118] Xiao Q., Riedesser J.E., Mulholland T., Li Z., Buchloh J., Albrecht P., Yang X., Li M., Venkatachalam N., Skabkina O. (2025). Combined MEK and PARP inhibition enhances radiation response in rectal cancer. Cell Rep. Med..

